# NeuroBase: A Web-Based Transcriptomic Resource and R Package for Cross-Disorder Analysis of Neurodegenerative and Neuropsychiatric Diseases

**DOI:** 10.1007/s12035-026-06169-0

**Published:** 2026-11-12

**Authors:** Alsamman M. Alsamman, Manar S. Ibrahim, Khaled H. Mousa, Saifeldeen M. Ibrahim, Morad M. Mokhtar, Anas M. Saedwi, Radwa Khaled, Achraf El Allali, Hatem Zayed

**Affiliations:** 1 Arthritis and Clinical Immunology Program, Oklahoma Medical Research Foundation, Oklahoma City, OK 73104 USA; 2 Agricultural Genetic Engineering Research Institute (AGERI), Agricultural Research Center (ARC), Giza, Egypt; 3 Computational and Bioinformatics Lab, Biomedical Science Program, University of Science and Technology, Zewail City, Giza, Egypt; 4 Clinical Omics and Informatics Unit, Neuroscience Institute, University of Cape Town, Cape Town, South Africa; 5 Chemical and Biochemical Sciences – Green Process Engineering, Mohammed VI Polytechnic University (UM6P), Ben Guerir, 43150 Morocco; 6 Translational and Applied Science Hub, Faculty of Medicine, Ain Shams University, Cairo, 11566, Egypt; 7 Bioinformatics Laboratory, College of Computing, Mohammed VI Polytechnic University (UM6P), Ben Guerir, 43150 Morocco; 8 Department of Biomedical Sciences, College of Health Sciences, QU Health, Qatar University, PO Box 2713 Doha, Qatar

**Keywords:** Neurodegenerative disorders, Neuropsychiatric disorders, Bulk RNA sequencing, Differential gene expression, Cross-disorder analysis, Transcriptomic database, Principal component analysis, R package, Reproducibility, Data resource

## Abstract

Neurodegenerative and neuropsychiatric disorders, including Alzheimer’s disease, autism spectrum disorder, bipolar disorder, Parkinson’s disease, and schizophrenia, are highly prevalent and biologically complex conditions that remain difficult to diagnose and treat. This study aimed to develop NeuroBase, a web-based transcriptomic resource that supports exploratory comparison of shared and disorder-specific gene expression features across these disorders. Fourteen human brain bulk RNA sequencing datasets (262 total samples) were processed using a standardized pipeline, followed by differential gene expression analysis with DESeq2, edgeR and limma-voom for broad exploratory discovery, complemented by a multi-method sensitivity analysis to define a method-concordant core gene set, protein–protein interaction network analysis using STRING with downstream exploration in Cytoscape, and functional enrichment analysis. Principal component analysis and clustering-based visualizations were used to summarize similarities and differences in transcriptomic patterns among disorders. Differential expression analysis identified dataset-level significant genes within each disorder using DESeq2 for one dataset per disorder (FDR ≤ 0.05), including 38 genes in Alzheimer’s disease, 6,704 in autism spectrum disorder, 3,559 in bipolar disorder, 687 in Parkinson’s disease, and 2,750 in schizophrenia; a stricter multi-method intersection analysis yielded method-concordant Core Intersection sets in the most informative datasets (983 core DEGs in autism spectrum disorder, 505 in bipolar disorder, 281 in schizophrenia, and 234 and 151 in two Parkinson’s disease datasets; only two core genes (SPP1 and CHST3) were found in Alzheimer’s disease). A unified strict-threshold reanalysis (FDR ≤ 0.05, |log₂FC|≥ 1) revealed disorder-level DEG burdens ranging from 34 (Alzheimer’s disease) to 2,812 (autism) deduplicated genes, but only 24 genes were reproducible across independent cohorts of the same disorder, highlighting the limited cross-cohort reproducibility characteristic of bulk brain transcriptomics. Enrichment analyses identified significantly enriched signaling pathways in autism spectrum disorder and nominally enriched synapse-related pathways in Alzheimer’s disease. Representative high-signal genes included SPP1 in Alzheimer’s disease, NRGN in one Parkinson’s disease dataset, and BEST1 in autism spectrum disorder. Cross-disorder comparison identified 465 differentially expressed genes shared between the neurodegenerative and neuropsychiatric disorder groups, and principal component analysis showed that sample variation was dominated by dataset and tissue of origin. NeuroBase is a publicly accessible, web-based transcriptomic database and companion R package (neurobaseR) that enables standardized, cross-disorder exploration of bulk brain gene expression profiles across neurodegenerative and neuropsychiatric disorders, supporting comparative analysis, programmatic data access, and hypothesis generation through interactive visualization and pathway-level summaries.

**Supplementary Information:**

The online version contains supplementary material available at https://doi.org/10.1007/s12035-026-06169-0.

## Introduction

Neurodegenerative and neuropsychiatric disorders (ND-NPs) represent a significant global health burden, impacting hundreds of millions of individuals worldwide (GBD 2016 Neurology Collaborators 2019; GBD 2019 Mental Disorders Collaborators 2022). Given their widespread prevalence and substantial economic and societal impact, understanding the underlying mechanisms of these disorders is crucial. These conditions profoundly affect multiple aspects of human life, including social interactions, physical health, and daily functioning. The complexity of ND-NPs has led to challenges in their clinical characterization and biological interpretation. Symptoms and behavioral changes often disrupt interpersonal relationships, leading to strained interactions and isolation (Ahmed et al. 2023). Therefore, understanding these disorders is essential for developing improved diagnostic frameworks and therapeutic strategies (Baker et al. 2020).

Alzheimer’s disease (AD), Autism Spectrum Disorder (ASD), Bipolar Disorder (BD), Parkinson’s disease (PD), and Schizophrenia (SCZ) are complex, multifactorial conditions driven by the interaction of multiple genes and environmental factors. These disorders contribute significantly to the global disease burden, resulting in substantial morbidity and mortality (GBD 2016 Neurology Collaborators 2019; GBD 2019 Mental Disorders Collaborators 2022). The complexity and diverse symptomatology of these disorders pose significant challenges in establishing definitive diagnostic criteria and universally accepted definitions across clinical and research settings. Despite advances in understanding the biological basis of mental disorders, genetic data for many major conditions remain limited (Li 2022), exhibiting a highly polygenic architecture involving multiple genes, variants, and environmental factors interacting across biological scales (Wang et al. 2023).

Although significant progress has been made in identifying genetic risk factors for disorders like schizophrenia and autism through large-scale genomic studies (Chaste and Leboyer 2012; Owen 2023), the intricate genetic mechanisms underlying these conditions remain poorly understood. This knowledge gap hinders the development of robust biomarkers and mechanism-informed therapeutic approaches. Comprehensive exploration of the genetic underpinnings is critical for unveiling novel therapeutic targets and advancing more effective, tailored interventions (Owen 2023).

RNA sequencing is a pivotal tool in understanding complex diseases, enabling the discovery of potential biomarkers and the elucidation of molecular mechanisms. Differential gene expression analysis, in particular, identifies genes and regulatory pathways with altered expression patterns in affected individuals compared to healthy controls. This approach enables systematic characterization of transcriptional dysregulation associated with ND-NPs, offering critical insights into the biological processes underlying these disorders and advancing our understanding of their pathogenesis (Li et al. 2022). Given the current heterogeneity and limited cross-disorder availability of single-cell transcriptomic datasets, this study focuses on bulk RNA-seq data, which remains the most broadly available and methodologically comparable approach for cross-disorder transcriptomic analysis. This study aims to construct a publicly accessible, web-based transcriptomic database (NeuroBase) of gene expression profiles associated with neurodegenerative and neuropsychiatric disorders. All downstream analyses were performed to populate, validate, and demonstrate the utility of the platform, enabling users to explore shared and disorder-specific differentially expressed genes and transcriptomic patterns across conditions.

## Materials and Methods

### Data Sources and Preprocessing

The transcriptome datasets utilized in this study were obtained from the publicly available Gene Expression Omnibus (GEO) database (https://www.ncbi.nlm.nih.gov/geo/), which provided RNA-Seq datasets of human brain samples. A total of 14 RNA-Seq transcriptomic datasets from five brain diseases were selected for inclusion in this study. Datasets were selected based on disease relevance, availability of human brain bulk RNA-seq data, and compatibility with a unified processing pipeline. The selection prioritized cross-disorder comparability rather than dataset size or cell-type resolution, enabling consistent transcriptome-level integration within the NeuroBase platform.

Specifically, three datasets were analyzed for AD, two for ASD, three for BD, three for PD, and three for SCZ (Table 1). To generate the final output—the count table for differential gene expression (DGE) analysis—RNA-seq data processing was performed using high-performance computing (HPC).

**Table 1:** Summary of datasets employed in the study

Disease	Dataset	Samples	Control	Patient
Alzheimer	PRJNA232669	17	8	9
	PRJNA688060	7	4	3
	PRJNA767074	10	5	5
Autism	PRJNA143369	12	6	6
	PRJNA869106	39	19	20
Bipolar	PRJNA318642	36	18	18
	PRJNA321439	16	8	8
	PRJNA555337	22	11	11
Parkinson	PRJNA417394	32	18	14
	PRJNA494382	17	8	9
	PRJNA563007	16	8	8
Schizophrenia	PRJNA268948	11	6	5
	PRJNA358689	8	4	4
	PRJNA551698	19	9	10
Total	14	262	132	130

PRJNA551698 and PRJNA555337 share nine control samples, as deposited in the public repository; the 262 samples therefore comprise 253 unique libraries

The key steps involved in this process were as follows: i) Data Download: FASTQ files were acquired from the Sequence Read Archive (SRA) (Leinonen et al. 2011), which contained raw sequencing reads generated by an Illumina sequencer. ii) Quality Control: quality-based trimming of the raw reads was performed using Sickle (Joshi and Fass 2011), an adaptive trimming tool for fastq files. Sickle accepts single-end files and two paired-end files (forward and reverse) as input and generates two trimmed paired-end files. Reads were trimmed using a minimum quality threshold of 20 (-q 20), with the quality encoding specified by the -t option. iii) Read Alignment: the trimmed reads were aligned to the reference genome using STAR (Spliced Transcripts Alignment to a Reference) (Dobin et al. 2013). The alignment process in STAR consists of two steps: (a) Creating a genome index using the human reference genome (GRCh38) and a GTF file obtained from Ensembl (https://ftp.ensembl.org/pub/release-109/gtf/homo_sapiens/), (b) Mapping the reads to the genome. Several parameters were utilized, including runThreadN (utilizing 20 cores), readFilesIn (fastq files generated from the Sickle step), genomeDir (the path for the reference genome and GTF file), outFileNamePrefix (the prefix for all output files), and outSAMunmapped (specifying how to handle unmapped reads). IV) File Conversion: Samtools was employed to convert the SAM alignment files to BAM format, which involved using the “view” command and sorting the resulting BAM files (Danecek et al. 2021). Finally, V) Gene Expression Counting: the reads were aligned to a genome annotation using HTSeq-count (Anders et al. 2015). Specific parameters were employed to determine the gene expression count in each sample. A sorted alignment BAM file and a GTF file were used. The count of gene expression for each sample in each experiment was obtained to perform the analysis of differential gene expression. By following this comprehensive methodology, we processed the raw RNA-Seq data and obtained the necessary gene expression counts for subsequent analysis of gene expression differences across the studied brain diseases.

### Differential Expression Analysis

The differential gene expression (DGE) analysis was conducted using the R/Bioconductor package DESeq2 v1.40 (Love et al. 2014) under R 4.3. For the standard tier, each dataset was also analyzed with edgeR (Robinson et al. 2010) and limma-voom (Law et al. 2014); genes with FDR ≤ 0.05 in at least one method were retained, and the lowest FDR across methods is reported. Because this tier is the union of three methods, its overall false discovery rate may exceed 5%. The standard tier defines the outputs available in NeuroBase; the per-disorder DEG counts and Venn analyses reported in this article are instead based on DESeq2 alone, for one dataset per disorder (see Results). Output files were generated, providing information such as mean gene expression across replicates for each sample, log2 fold-change (LFC), p-value, and adjusted p-value (FDR). To identify significant genes, a false discovery rate (FDR) threshold of 0.05 was applied, and LFC was used to determine upregulated and downregulated genes (McDermaid et al. 2019). To evaluate the impact of normalization methods on the DE analysis, the data was normalized. Then, genes with average counts lower than 10 were filtered out using the DESeq2 package. A minimum mean count threshold was applied to reduce low-count noise and improve the robustness of differential expression estimates; consequently, some low-expression transcripts may not be retained, consistent with standard practices in bulk RNA-seq exploratory analyses. Subsequently, the data integrity was validated through various plots, including box plots and density plots of the normalized read counts. To assess analytical robustness, a parallel sensitivity analysis was performed across six methods: standard DESeq2 Wald test, likelihood ratio test (LRT), whose p-values were paired with the apeglm-shrunken log2 fold changes of the Wald fit (Zhu et al. 2019), limma-voom with quality weights (Law et al. 2014; Ritchie et al. 2015), SVA-corrected DESeq2 (Leek et al. 2012), RUVSeq-normalized DESeq2 (Risso et al. 2014), and a Wald filter at FDR < 0.01 and |log₂FC| > 1. Of these, five represent distinct analytical approaches (standard Wald, LRT, limma-voom, SVA-corrected, and RUVSeq-corrected); this Wald filter is a stricter re-application of the Wald test rather than a separate approach, and is therefore not included in the comparison of five analytical approaches in Fig. 2C; it provides the Wald criterion of the Core Intersection. A Core Intersection gene set was defined as genes with FDR < 0.05 and |log2FC| > 1 in both the LRT and the RUVSeq-corrected analyses and with FDR < 0.01 and |log2FC| > 1 in the Wald test, a threshold more stringent than the per-dataset strict tier, representing a method-concordant subset. All |log2FC| thresholds in this analysis were applied to apeglm-shrunken estimates (from the Wald fit for the LRT and Wald FDR < 0.01 criteria, and from the RUVSeq-adjusted model for the RUVSeq criterion). Standard-tier results (FDR ≤ 0.05 in DESeq2, edgeR or limma-voom) are reported as primary outputs in NeuroBase to maximize discovery breadth for user-level filtering; Core Intersection results are provided as a method-concordant reference set in the Supplementary Material; they have not been validated in independent cohorts. The sensitivity analysis, the Core Intersection and the strict-tier reanalysis (Fig. 2; Supplementary Tables S13–S14) come from a later, harmonized run of the pipeline on the same datasets, with its own gene filtering. The per-disorder DEG tables, counts and Venn analyses (Supplementary Tables S1–S7) come from the original DESeq2 analysis of each dataset, so a Core Intersection gene is not necessarily listed in Supplementary Table S7.

In addition to the Core Intersection consensus set, a unified per-dataset strict-threshold re-analysis was performed by applying a dual cutoff (FDR ≤ 0.05 and |log₂FC|≥ 1) to Standard Wald test results from a single harmonized pipeline run, independently for each of the 14 datasets. Cross-dataset reproducibility was assessed by requiring genes to reach this threshold in two or more independent datasets of the same disorder in a consistent direction; disorder-level burden was reported as deduplicated gene counts (a gene significant in multiple datasets of the same disorder counted once). This analysis quantified the proportion of up- and down-regulated genes per dataset and per disorder, and identified genes showing consistent direction of dysregulation across independent datasets within the same disorder. This dataset-level stringent reanalysis is reported separately from the Core Intersection set, as it reflects a single-threshold criterion applied per dataset rather than a multi-method consensus, and is intended to characterize the magnitude and reproducibility of transcriptomic signal across independent cohorts within NeuroBase.

To explore sample relationships and validate variability within each condition, Principal Component Analysis (PCA) was performed using the PCAtools package (Blighe et al. 2019). This within-condition PCA helped assess clustering and detect potential outliers in individual differential expression analyses. Gene expression data was also visualized using heatmaps, which, when combined with clustering methods, enabled the grouping of genes and/or samples based on their expression patterns. This approach aids in identifying commonly regulated genes and biological signatures associated with specific diseases. In this analysis, the R package ggplot2 was employed for heatmap visualization (Wickham 2016). Additionally, a volcano plot, a crucial visualization tool in differential analysis, was used to assess the overall significance of upregulated and downregulated genes (Supplementary Figs. S1–S5).

Furthermore, the R package ggVennDiagram was utilized to generate Venn diagrams comparing the DEG sets of one dataset per disorder across the five disorders, facilitating the exploratory identification of shared and unique genes among the diseases and providing insights into their relationships (Gao et al. 2021). Subsequently, the expression patterns of the 20 genes listed in four of the five per-disorder DEG tables (Supplementary Tables S1–S5, which list all 38 AD DEGs and, for ASD, BD, PD and SCZ, only the DEGs with FDR < 0.015), which are a subset of the 51 genes shared by four disorders in the Venn analysis (Supplementary Table S7), were investigated to get insights into whether these genes could also share similar expression patterns across these disorders.

To minimize technical confounding while maintaining analytical consistency, differential expression analyses were performed separately within each dataset using case/control contrasts, without pooling raw counts across studies. False discovery rate (FDR) adjustment was applied to control for multiple testing in transcriptome-wide analyses; more conservative corrections and independent cohort validation were not performed, as differential expression results are intended as exploratory outputs within the NeuroBase resource. Biological covariates such as age, sex, postmortem interval, and RNA integrity were not consistently available across datasets and therefore were not included in the differential expression models; sex-linked genes (for example XIST, TSIX and KDM5D) may therefore appear as differentially expressed because of unequal sex ratios between groups. Differential expression analyses were performed separately within each dataset without pooling samples across studies; therefore, batch effects across datasets were not explicitly modeled, as NeuroBase is intended for dataset-level exploratory analysis rather than cross-study meta-analysis.

### Protein–Protein Interaction Analysis (PPI)

To investigate the interactions between the top significant genes in each disease and to examine gene correlations across all five conditions, we selected significant genes identified through our DGE analysis in all datasets of each disorder, defined by an adjusted p-value (FDR) ≤ 0.05 in DESeq2, edgeR or limma-voom or, in datasets with few such genes, by a nominal p < 0.001. These input lists therefore differ from the per-disorder DEG sets used for the counts and Venn analyses. Protein–protein interaction (PPI) analysis was performed using the STRING database v11.5 (Szklarczyk et al. 2021; accessed May 2023) with the multiple proteins search option, retaining default advanced settings. The full-string network type was used with a required score of medium confidence (0.400) and a medium FDR stringency (5%). The resulting PPI networks were imported into Cytoscape (Shannon et al. 2003) for further analysis. In Cytoscape, interacting proteins for each disease were identified and clustered into two groups based on their expression direction (up- or down-regulated) and logFC values, as specified in the DGE analysis results. Network clustering was performed in Cytoscape using the MCODE algorithm (Bader and Hogue 2003) to identify densely connected gene modules, which are exploratory and are not presented in this article.

### Gene Enrichment Analysis

Functional enrichment (over-representation) analysis was used to identify biological pathways over-represented among the DEGs (Xie et al. 2021). Gene sets were drawn from Gene Ontology (GO) and Kyoto Encyclopedia of Genes and Genomes (KEGG) pathways (Islam et al. 2022). Through the analysis of these gene sets, enrichment analysis enables insights into the underlying biological mechanisms and their associations with specific conditions or phenotypes. The utilization of the STRING database and the Enrichr online bioinformatics tool (Xie et al. 2021), developed by the Ma’ayan Lab, facilitated Gene Ontology enrichment studies encompassing biological processes, cellular components, and molecular functions, along with KEGG pathway enrichment analysis. This involved the up-regulated and down-regulated gene sets derived from the resulting differentially expressed genes (DEGs).

### Principal Component Analysis

To explore overarching patterns across disorders, Principal Component Analysis (PCA) was performed on patient samples from all conditions by merging their count data into a unified matrix, explicitly excluding control samples. This analysis focused on uncovering similarities or differences in gene expression profiles across disorders. The count matrix was first filtered to retain genes with a mean count greater than 10, then normalized using DESeq2 size factors (estimateSizeFactors on a DESeqDataSet object), followed by Count Per Million (CPM) calculation and log2 transformation of the normalized counts.

PCA was then conducted using the prcomp function in R. This cross-disorder PCA is exploratory: because no batch correction was applied, sample groupings may reflect study origin, brain region and technical factors as well as disease.

### neurobaseR: R Package for Programmatic Data Access

To enable reproducible, programmatic access to NeuroBase data, we developed neurobaseR, an R package available at https://github.com/AlsammanAlsamman/neurobaseR. The package provides direct retrieval of raw count matrices and differential expression results for all 14 datasets included in this study. Both the standard DEG results (FDR ≤ 0.05 in DESeq2, edgeR or limma-voom) and the Core Intersection method-concordant gene sets are accessible through unified query functions, allowing users to select their preferred stringency level for downstream analysis. Visualization functions within neurobaseR reproduce the key figures described in this study — including volcano plots, heatmaps, and cross-disorder comparison plots — enabling users to regenerate or customize outputs within their own R environment.

## Results

### Sample Characteristics and Clustering

All results presented in Sections "Exploratory Identification of the Top Significantly Differentially Expressed Genes (DEGs) Across Five Neurological Diseases"–"Protein–Protein Interaction Analysis" are intended to illustrate the scope and functionality of NeuroBase and should be interpreted as exploratory summaries rather than definitive disease-specific findings. We analyzed 14 RNA-Seq datasets from patients with AD, ASD, BD, PD, and SCZ (see Table 1 for details). To ensure comparability across datasets, all samples were processed using a standardized RNA-Seq pipeline to generate harmonized gene expression profiles for integration into NeuroBase. Principal component analysis (PCA) was applied to provide an exploratory, low-dimensional representation of samples across disease groups based on transcriptomic similarity (see Methods for details).

### Exploratory Identification of the Top Significantly Differentially Expressed Genes (DEGs) Across Five Neurological Diseases

**Fig. 1:**
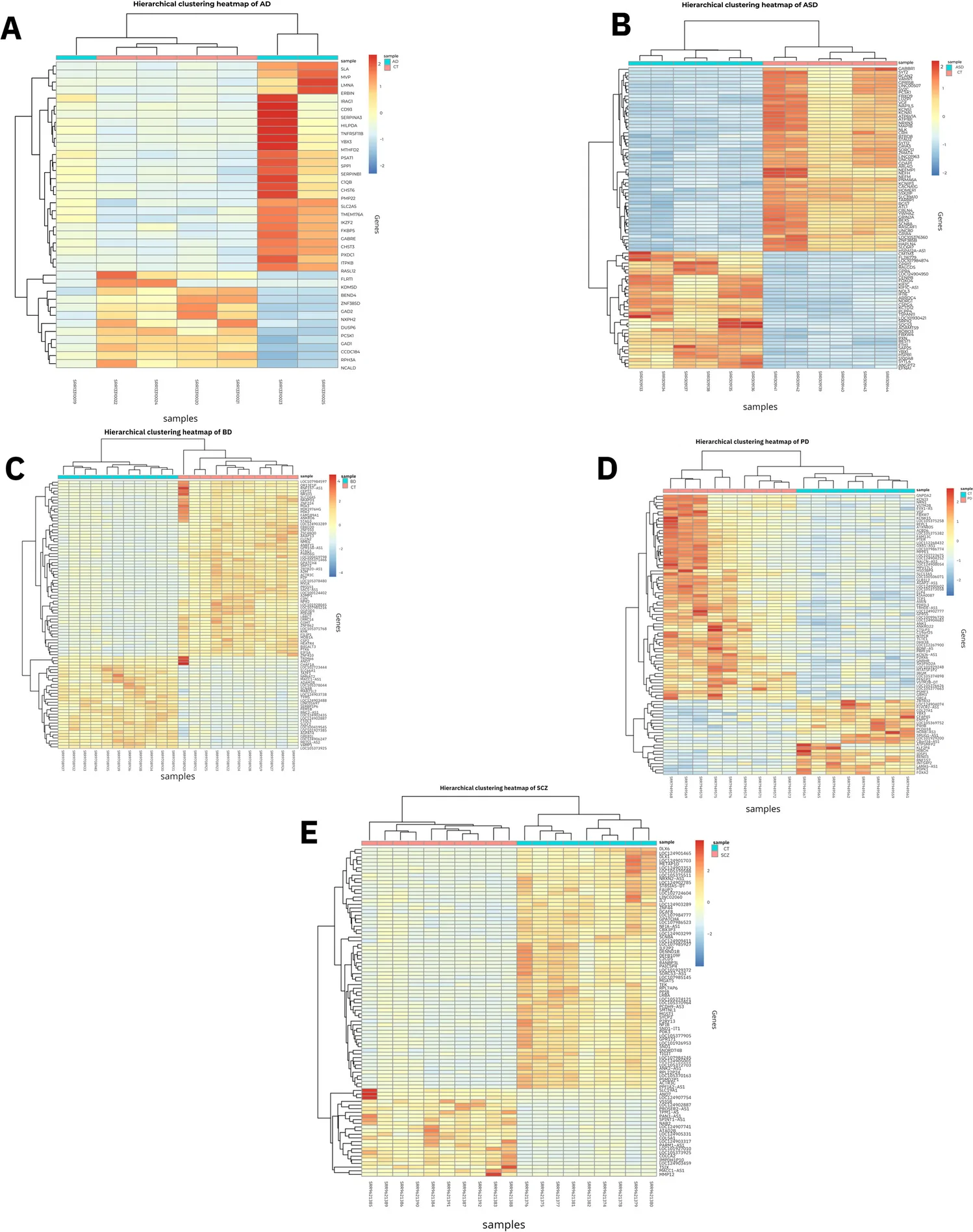
Hierarchical clustering of the top significantly differentially expressed genes (FDR < 0.05) across five neurological disorders. Low expression levels are represented by blue pixels, while high expression levels are shown in red. Each row corresponds to a differentially expressed gene. Panels: (**A**) Alzheimer’s disease (AD), (**B**) Autism spectrum disorder (ASD), (**C**) Bipolar disorder (BD), (**D**) Parkinson’s disease (PD), and (**E**) Schizophrenia (SCZ)

**Fig. 2:**
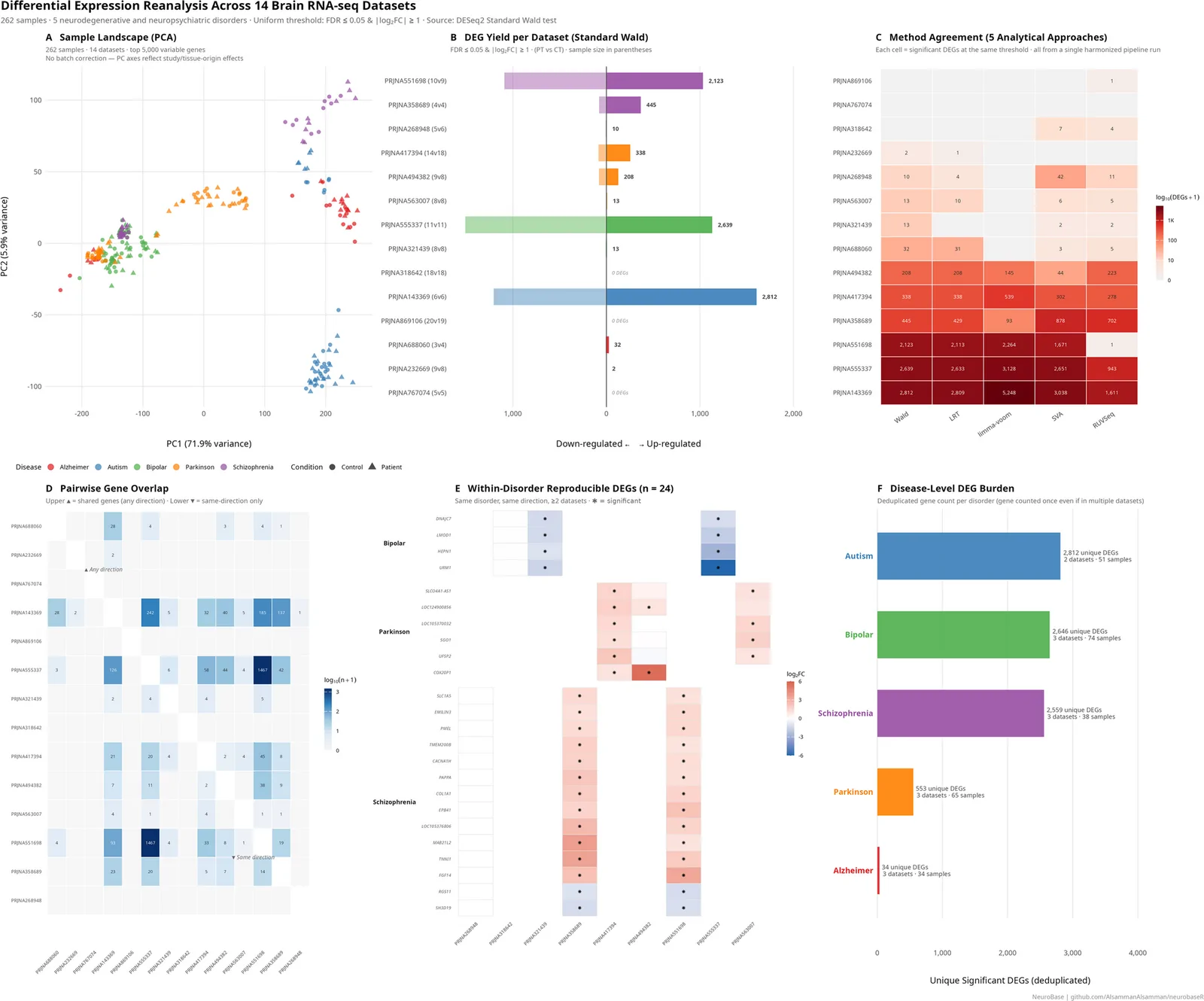
Comprehensive strict-threshold differential expression reanalysis across 14 brain RNA-seq datasets spanning five neurological disorders. All panels are based on a single harmonized pipeline applied to patient (PT) versus control (CT) comparisons across 262 samples, with a uniform threshold of FDR ≤ 0.05 and |log₂FC|≥ 1. Panels B, D, E and F use the DESeq2 Standard Wald test; panel C compares five analytical approaches at the same threshold. **A** Principal component analysis of all 262 samples based on the top 5,000 most variable genes, without batch correction; points are colored by disorder and shaped by condition (triangle = patient, circle = control). Samples cluster primarily by dataset and tissue origin rather than by condition, reflecting study-level batch and tissue-source effects. This view is computed differently from the disorder-level PCA in Fig. 6 (Section "Principal Component Analysis"), which uses patient samples only; the two are complementary. **B** Diverging bar plot of up-regulated (right) and down-regulated (left) DEGs per dataset; per-dataset sample sizes (patients × controls) are shown in parentheses. DEG yield varies by more than three orders of magnitude across cohorts. **C** Heatmap of the number of significant DEGs detected by each of five analytical approaches (Standard Wald, LRT, limma-voom, SVA-corrected, RUVSeq-corrected) across all 14 datasets, with color intensity corresponding to log₁₀-transformed DEG counts. Largely concordant detection in high-signal datasets (except PRJNA551698 under RUVSeq) suggests that results are not mainly method-specific artifacts. **D** Pairwise gene overlap between datasets; the upper triangle shows total shared DEGs (any direction) and the lower triangle shows same-direction shared DEGs only, revealing frequent directional disagreement in cross-disorder overlaps. PRJNA555337 (BD) and PRJNA551698 (SCZ) share nine control samples (Table 1), which may partly inflate the overlap between these two datasets. **E** Heatmap of log₂ fold-change values for the 24 genes reaching significance in two or more independent datasets of the same disorder in a consistent direction, grouped by disorder; asterisks mark datasets where each gene reached significance. **F** Deduplicated total significant DEG counts per disorder, in which a gene significant in multiple datasets of the same disorder is counted once

**Fig. 3:**
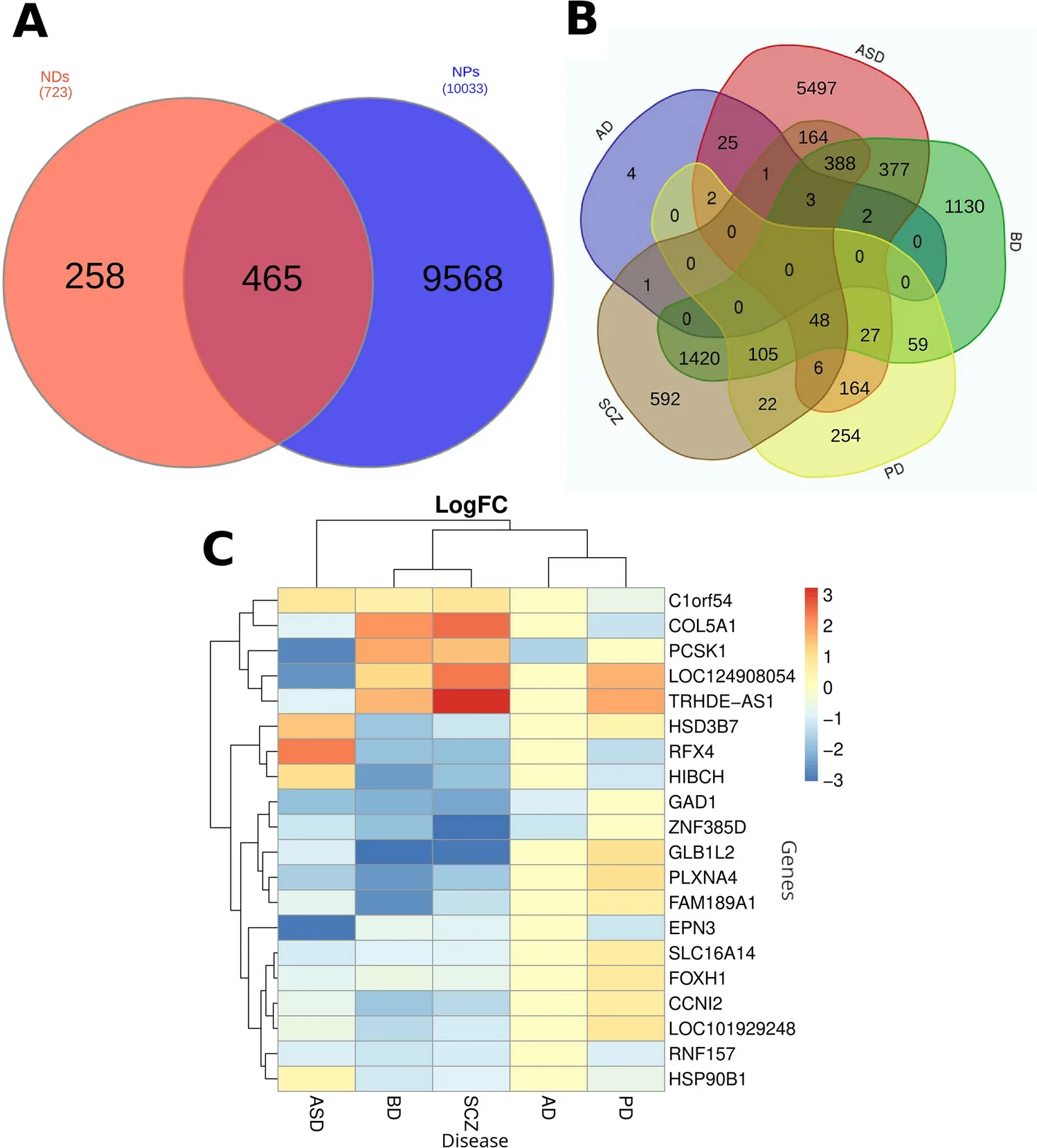
Overlap and expression patterns of differentially expressed genes (DEGs) (FDR < 0.05) across neurological disorders. **A** Venn diagram illustrating the overlap of DEGs between Neurodegenerative disorders (NDs; shown in red) and Neuropsychiatric disorders (NPs; shown in blue). Numbers indicate unique and shared DEGs. NRGN, PDE1B, and GABRA2, discussed in the text for PD, were identified in dataset PRJNA563007 and are not included in the PD set. **B** Venn diagram showing the overlap of DEGs across all five individual disorders (AD, ASD, BD, PD, SCZ). Numbers indicate the count of DEGs unique to each disorder or shared between specific combinations; the 465 genes reported in the text are the ND–NP intersection shown in panel **A**. **C** Heatmap displaying the expression patterns of the 20 genes, among the 51 shared by four disorders (Supplementary Table S7), that are listed in four of Supplementary Tables S1–S5. Rows represent genes, columns represent disorders/conditions. Color indicates log fold change (logFC; red = upregulated, blue = downregulated); the disorder in which a gene is not significant is shown as 0. All genes in C are significant in both BD and SCZ, whose datasets share nine control samples (Table 1), which may partly inflate their overlap

Differential expression results are reported at three stringency tiers: a standard tier (FDR ≤ 0.05 in DESeq2, edgeR or limma-voom) for broad discovery, a per-dataset strict tier (FDR ≤ 0.05 and |log₂FC|≥ 1) for effect-size–filtered signals, and a method-concordant Core Intersection tier. Unless otherwise stated, the per-disorder counts below and the Venn analyses (Fig. 3; Supplementary Tables S6–S7) are based on DESeq2 alone for one dataset per disorder (AD, PRJNA688060; ASD, PRJNA143369; BD, PRJNA555337; PD, PRJNA494382; SCZ, PRJNA551698), not on the three-method standard tier that defines the NeuroBase outputs. A gene was counted if its DESeq2 FDR was below 0.05. The complete gene sets behind these counts are listed in Supplementary Table S7; Supplementary Tables S2–S5 list only the genes with FDR < 0.015. FDR values quoted for individual genes are the DESeq2 values listed in Supplementary Tables S1–S5 or, for PRJNA563007, the DESeq2 values of that dataset. In Alzheimer’s disease (AD), we identified 38 genes with significant differential expression (Supplementary Table S1; Fig. 1A, Supplementary Fig. S1). Significant genes included SPP1 (FDR = 2.03 × 10^–10^), CHST3 (FDR = 4.80 × 10^–4^), and BEND4 (FDR = 0.0097). In Autism spectrum disorder (ASD), differential expression analysis identified 6,704 significant genes (Supplementary Table S2; Fig. 1B, Supplementary Fig. S2). This high number of differentially expressed genes is presented as a transcriptome-wide descriptive pattern reflecting the biological heterogeneity of ASD, rather than as a definitive disease-specific gene signature. Among the most notable were BEST1 (FDR = 9.75 × 10^–30^), GABBR1 (FDR = 6.59 × 10^–14^), and HOMER1 (FDR = 5.75 × 10^–24^). In Bipolar disorder (BD), we identified 3,559 significantly differentially expressed genes (Supplementary Table S3; Fig. 1C, Supplementary Fig. S3). FILIP1 (FDR = 6.1 × 10^–17^) was among the most significant genes, and SCN9A (FDR = 4.83 × 10^–4^) was also differentially expressed. In Parkinson’s disease (PD), a total of 687 genes were significantly differentially expressed (Supplementary Table S4; Fig. 1D, Supplementary Fig. S4). In a second PD dataset (PRJNA563007), which is not part of this count, the top candidates included NRGN (FDR = 9.74 × 10^–9^), PDE1B (FDR = 2.73 × 10^–8^), and GABRA2 (FDR = 2.34 × 10^–8^). In Schizophrenia (SCZ), 2,750 genes showed significant differential expression (Supplementary Table S5; Fig. 1E, Supplementary Fig. S5). DLX6 was among the most significant genes (FDR = 6.13 × 10^–15^). To contextualize these counts, a multi-method sensitivity analysis identified method-concordant Core Intersection gene sets in nine datasets, five of which contained more than 100 genes: 983 core DEGs in PRJNA143369 (ASD), 505 in PRJNA555337 (BD), 281 in PRJNA358689 (SCZ), 234 in PRJNA417394 (PD), and 151 in PRJNA494382 (PD); the remaining four yielded 1–3 core genes each (Supplementary Table S13), including two (SPP1 and CHST3) in the AD dataset PRJNA688060, and the other two AD datasets yielded none, which may reflect small cohort sizes, tissue heterogeneity or technical factors. PRJNA551698 (SCZ) yielded a single core gene, the X-linked TSIX, because the RUVSeq-corrected analysis retained almost no significant genes in this dataset. These core genes, available in Supplementary Table S13, are method-concordant candidates (supported by several statistical approaches within the same dataset) and are recommended as a starting point for independent validation studies. A unified strict-threshold reanalysis across all 14 datasets (FDR ≤ 0.05 and |log₂FC|≥ 1, applied to Standard Wald test results from a single harmonized pipeline) revealed substantial variability in transcriptomic burden both within and between disorders (Fig. 2). At the disorder level, deduplicated significant DEG counts were highest in Autism (2,812 unique DEGs across 2 datasets), followed by Bipolar disorder (2,646 across 3 datasets), Schizophrenia (2,559 across 3 datasets), Parkinson's disease (553 across 3 datasets), and Alzheimer's disease (34 across 3 datasets) (Fig. 2F). Per-dataset DEG yield spanned more than three orders of magnitude, ranging from ≤ 2 DEGs in four datasets (PRJNA318642, PRJNA767074 and PRJNA869106 with none; PRJNA232669 with two) to 2,812 in PRJNA143369 (Autism), with PRJNA551698 (Schizophrenia) and PRJNA555337 (Bipolar) also showing high yield (Fig. 2B). Across the five analytical approaches (Standard Wald, LRT, limma-voom, SVA-corrected, and RUVSeq-corrected), most high-signal datasets showed concordant detection (PRJNA551698, in which the RUVSeq-corrected analysis retained a single gene, is an exception), suggesting that these results are largely not driven by method-specific artifacts (Fig. 2C). However, cross-dataset reproducibility under this stringent criterion was limited: only 24 genes reached significance in two or more independent datasets of the same disorder in a consistent direction (Schizophrenia, 14; Parkinson's disease, 6; Bipolar disorder, 4; Autism and Alzheimer’s disease, 0; Fig. 2E and Supplementary Table S14). Pairwise overlap analysis further showed that genes shared between datasets of different disorders frequently disagreed in direction — for example, of 137 genes shared between PRJNA143369 (Autism) and PRJNA358689 (Schizophrenia), only 23 shared the same direction of change (Fig. 2D). Principal component analysis of all 262 samples, computed without batch correction, showed that samples clustered primarily by dataset and tissue origin rather than by patient–control status, reflecting strong study-level technical and biological heterogeneity across independent cohorts (Fig. 2A). This limited within-disorder reproducibility does not indicate an absence of disease-associated signal, but rather reflects small per-study cohort sizes, differences in brain region and postmortem handling, and the conservative nature of a single-threshold criterion, and directly motivates NeuroBase’s multi-tier reporting strategy.

### Cross-Condition Transcriptomic Overlaps Observed Among Neurological Diseases

The Venn diagram analysis revealed 465 differentially expressed genes (DEGs) shared between the neurodegenerative (NDs) and neuropsychiatric (NPs) disorder groups at an FDR < 0.05; these are not genes common to all five disorders (Figs. 3A, Supplementary Table S6). Supplementary Table S6 provides a detailed summary of shared and disorder-specific DEGs identified through the Venn diagram analysis, offering contextual support for the overlaps described here. These genes are significant in more than one disorder, although not always with the same direction of change. No gene was shared by all five disorders, and 51 genes were shared by four (Supplementary Table S7). Fig. 3C shows the 20 of these 51 genes that are listed in four of the per-disorder DEG tables (Supplementary Tables S1–S5), with disorder-dependent direction and magnitude of change. All 51 genes are significant in both BD and SCZ, whose datasets (PRJNA555337 and PRJNA551698) share nine control samples (Table 1); the overlap between these two disorders may therefore be partly inflated. Among the 20 genes in Fig. 3C, C1orf54 was upregulated in ASD, BD, and SCZ and modestly downregulated in PD. Another gene of interest, TRHDE-AS1, a cytoplasmic long non-coding RNA, was upregulated in SCZ, PD, and BD and downregulated in ASD. This gene displayed distinct disorder-specific expression patterns across the analyzed datasets. In contrast, ZNF385D was downregulated in all four disorders in which it was significant (SCZ, AD, BD, and ASD).

### Unique Transcriptomic Signatures Across Disorders

The analysis also revealed unique transcriptomic signatures for each disorder. AD presented 38 significant DEGs, four of which were unique, including DUSP6, FKBP5, and TNFRSF11B. PD showed 254 unique DEGs, including PDPK1. ASD exhibited the highest number of unique genes, with 5,497 exclusive DEGs, including GPR4. BD demonstrated 1,130 unique DEGs, including LONP1, while SCZ showed 592 unique DEGs, exemplified by TSTD3. These results highlight disorder-specific transcriptomic patterns alongside shared gene expression features across conditions (Figs. 3B, 3C; Supplementary Table S7).

### Biological Relevance and Enriched Pathways

**Fig. 4:**
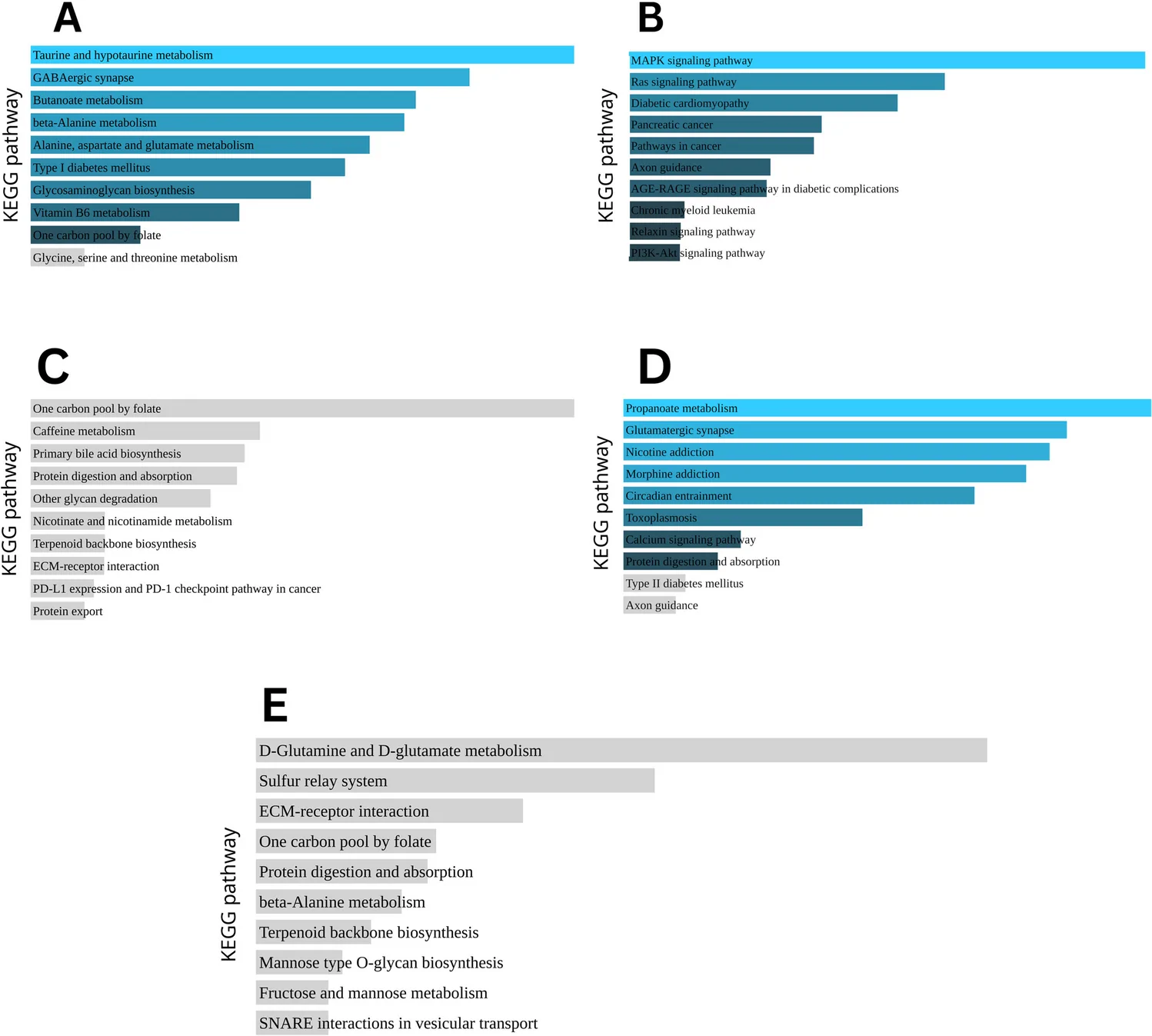
The top enriched biological pathways associated with significantly differentially expressed genes (DEGs) in each neurological disorder. Bar plots display the top 10 enriched pathways ranked by enrichment significance (bar length). Colored bars indicate nominal p < 0.05 (brighter color = smaller p) and grey bars are not significant. Only the ASD pathways reached adjusted p < 0.05. **A** Alzheimer’s disease (AD), (**B**) Autism spectrum disorder (ASD), (**C**) Bipolar disorder (BD), (**D**) Parkinson’s disease (PD), and (**E**) Schizophrenia (SCZ)

KEGG pathway enrichment analysis identified pathways reaching FDR < 0.05 only in ASD. For AD, the top-ranked pathways (nominal p < 0.05) were taurine and hypotaurine metabolism and GABAergic synapse signaling (Fig. 4A). In ASD, MAPK and RAS signaling pathways were identified as significantly enriched, with an FDR < 0.05 (Fig. 4B). However, for BD, PD, and SCZ, pathway enrichment analysis did not identify pathways reaching FDR < 0.05 (Figs. 4C, 4D, and 4E, respectively).

### Protein–Protein Interaction Analysis

**Fig. 5:**
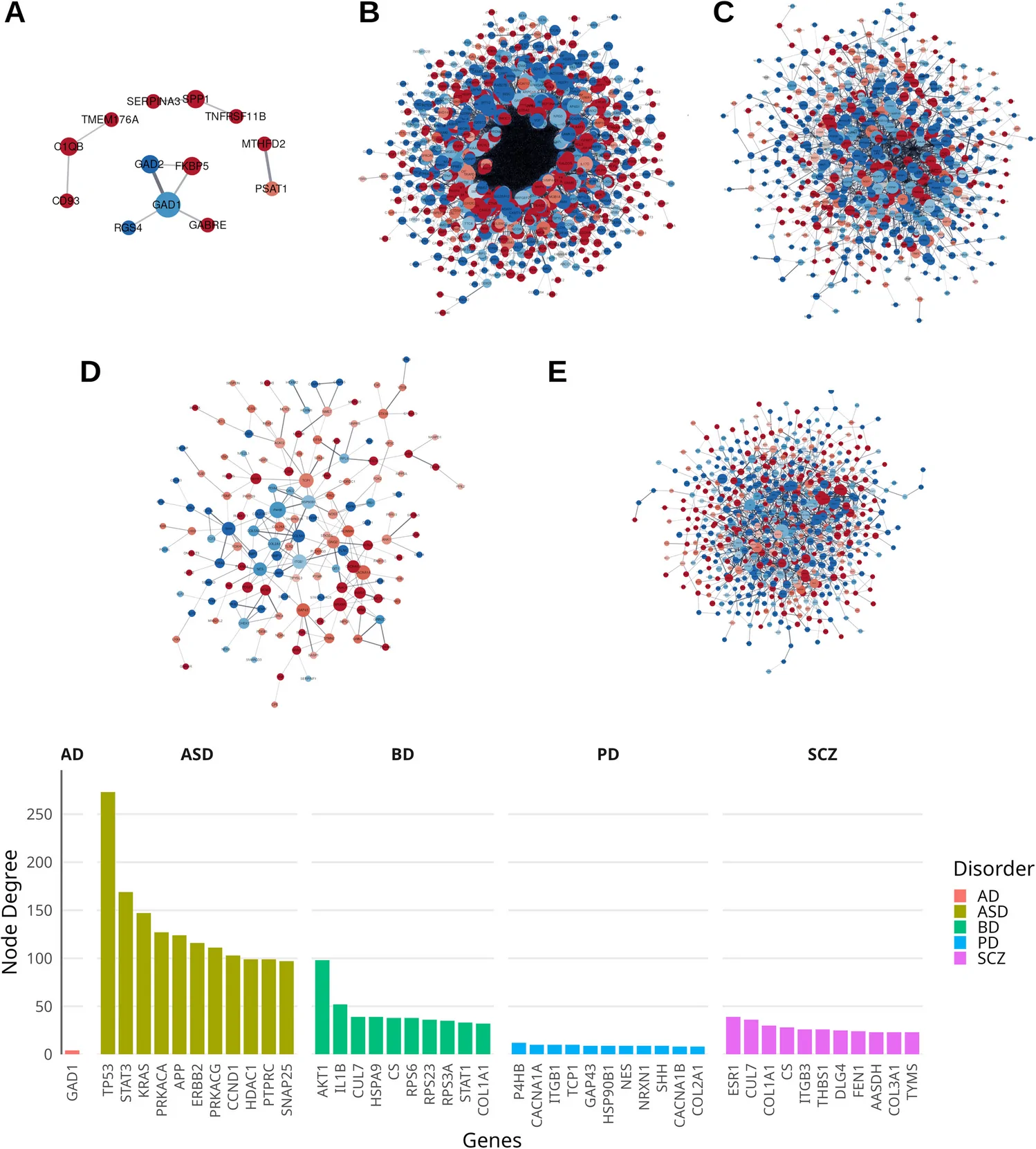
Protein–protein interaction (PPI) networks and hub gene analysis for differentially expressed genes (DEGs). Networks were constructed using the STRING database. Nodes represent proteins/genes, and edges represent STRING interactions supported by experimental, curated-database, predicted or text-mining evidence. Panels show the main interaction network component for DEGs in: (**A**) AD, (**B**) ASD, (**C**) BD, (**D**) PD, and (**E**) SCZ. Below the networks, a single bar plot shows the 10 or 11 most highly interactive genes from the ASD, BD, PD and SCZ networks and the most connected gene (GAD1) from the small AD network; node degrees for all genes are given in Supplementary Tables S8–S12. Gene names are on the X-axis, and the node degree is on the Y-axis. Bars are color-coded by disorder as indicated in the legend

The protein–protein interaction (PPI) networks for five neurological diseases were constructed to visualize gene connectivity patterns. In these networks, red nodes represent upregulated genes, while blue nodes indicate downregulated genes. Node size corresponds to the degree of connectivity (the number of interactions a protein has within the network), with larger nodes highlighting genes with higher interaction degrees (Fig. 5A–E). A bar plot highlights the genes with the highest node degrees for each disorder. ASD shows the highest number of genes with elevated node degrees, with TP53 and STAT3 among the most connected genes. BD ranks second, with AKT1 showing a high degree of connectivity within its network (Fig. 5). Detailed information on node degree values is provided in the supplementary materials (Tables S8–S12).

### Principal Component Analysis

**Fig. 6:**
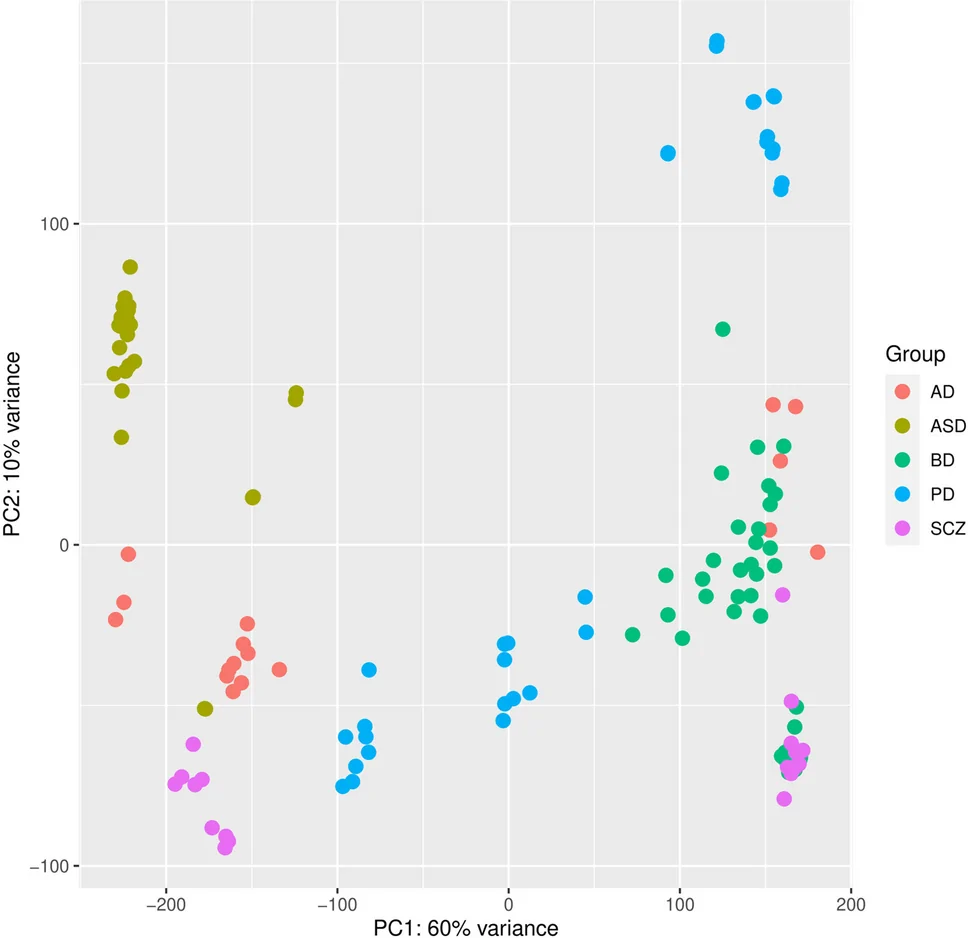
Principal Component Analysis (PCA) of gene expression profiles from samples across five neurological disorders (AD, ASD, BD, PD, and SCZ). Each point represents an individual patient sample (controls excluded; no batch correction), color-coded by disorder. The plot shows Principal Component 1 (PC1, X-axis, 60% variance explained) versus Principal Component 2 (PC2, Y-axis, 10% variance explained)

To explore the overall relationships between the gene expression profiles across the five neurological disorders and visualize the major sources of variation in the dataset, we performed Principal Component Analysis (PCA). This technique reduces the dimensionality of the data, allowing visualization of how samples group based on their global transcriptomic signatures. The analysis revealed that the first two principal components captured a substantial portion of the total variance present in the gene expression data. Specifically, the first principal component (PC1) explained 60% of the variance, representing the largest source of variation across samples. The second principal component (PC2) accounted for an additional 10% of the variance (Fig. 6). Together, these components capture 70% of the overall gene expression variability, providing an overview of sample distribution and variability across disorders.

The resulting PCA plot (Fig. 6) illustrates the distribution of samples based on these principal components. Because no batch correction was applied and each disorder is represented by only two or three datasets, these groupings largely reflect dataset and tissue of origin (Fig. 2A) and should not be read as disease-level similarity. The plot also showed relative proximities between certain disorders. Samples from AD and ASD exhibited closer positioning, particularly along PC1. Some AD samples were also positioned near the BD group. Partial overlap was observed between samples from SCZ, PD, and BD. In contrast, ASD samples formed a cluster that remained relatively distinct from BD samples in this two-dimensional projection.

The variance captured by these components is likely to reflect study-level technical and biological heterogeneity as well as any disease-related differences.

### NeuroBase: A Database for Neurodegenerative and Neuropsychiatric Gene Expression Profiling

**Fig. 7:**
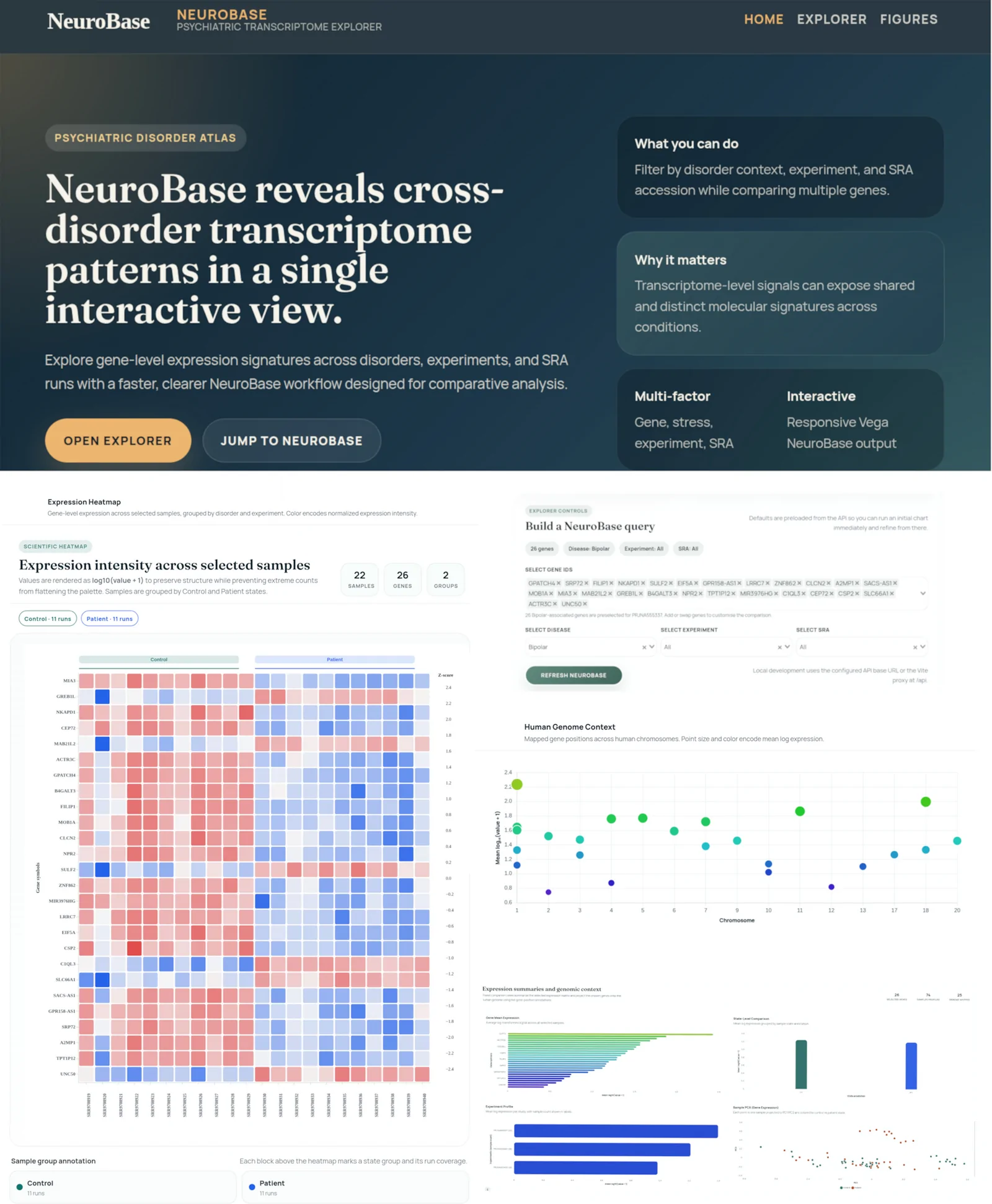
Screenshot of the NeuroBase web application interface. The platform provides a centralized transcriptomic database and analytical toolkit for cross-disorder exploration, including gene-level browsing, differential expression results, pathway/enrichment summaries, protein–protein interaction network visualization, and interactive plots across Alzheimer’s disease, Parkinson’s disease, autism spectrum disorder, bipolar disorder, and schizophrenia

Our work culminated in the development of NeuroBase, a centralized, web-accessible database consolidating all gene count and differential expression analyses performed in this study. NeuroBase houses transcriptome data from five major neurodegenerative and mental disorders, AD, ASD, BD, PD, and SCZ, and is designed to support exploratory analysis of gene expression patterns across disorders, providing a comprehensive resource for comparative transcriptomic exploration (Fig. 7).

The database catalogs up to 59,251 annotated human genes, of which 29,489 were tested for differential expression after count-based filtering, providing comprehensive transcriptomic coverage across the included datasets. Differential gene expression analyses were conducted using DESeq2, edgeR and limma-voom, identifying genes with significant expression changes across disorders. The database features intuitive heatmap visualizations, which enable users to explore shared and distinct gene expression patterns among conditions. These visualizations are interactive, enabling users to dynamically filter genes, select specific disorders, adjust display parameters, and explore expression patterns and PCA projections in real time through the web interface. NeuroBase is implemented using a modular web-based architecture with structured backend storage to support scalable data management and interactive querying. The platform provides user-driven features including dynamic filtering of genes and datasets, interactive visualization of expression profiles, pathway enrichment results, and PPI networks. While the current release focuses on transcriptomic data, the underlying architecture is designed to be extensible and compatible with future integration of additional omics layers and custom analytical workflows.

In addition to individual gene-level data, NeuroBase allows users to investigate biological pathways relevant to mental health disorders. The pathway exploration feature enables researchers to examine expression levels of selected genes or pathways across multiple disorders, facilitating the exploratory identification of common molecular mechanisms or disorder-specific pathways.

**Fig. 8:**
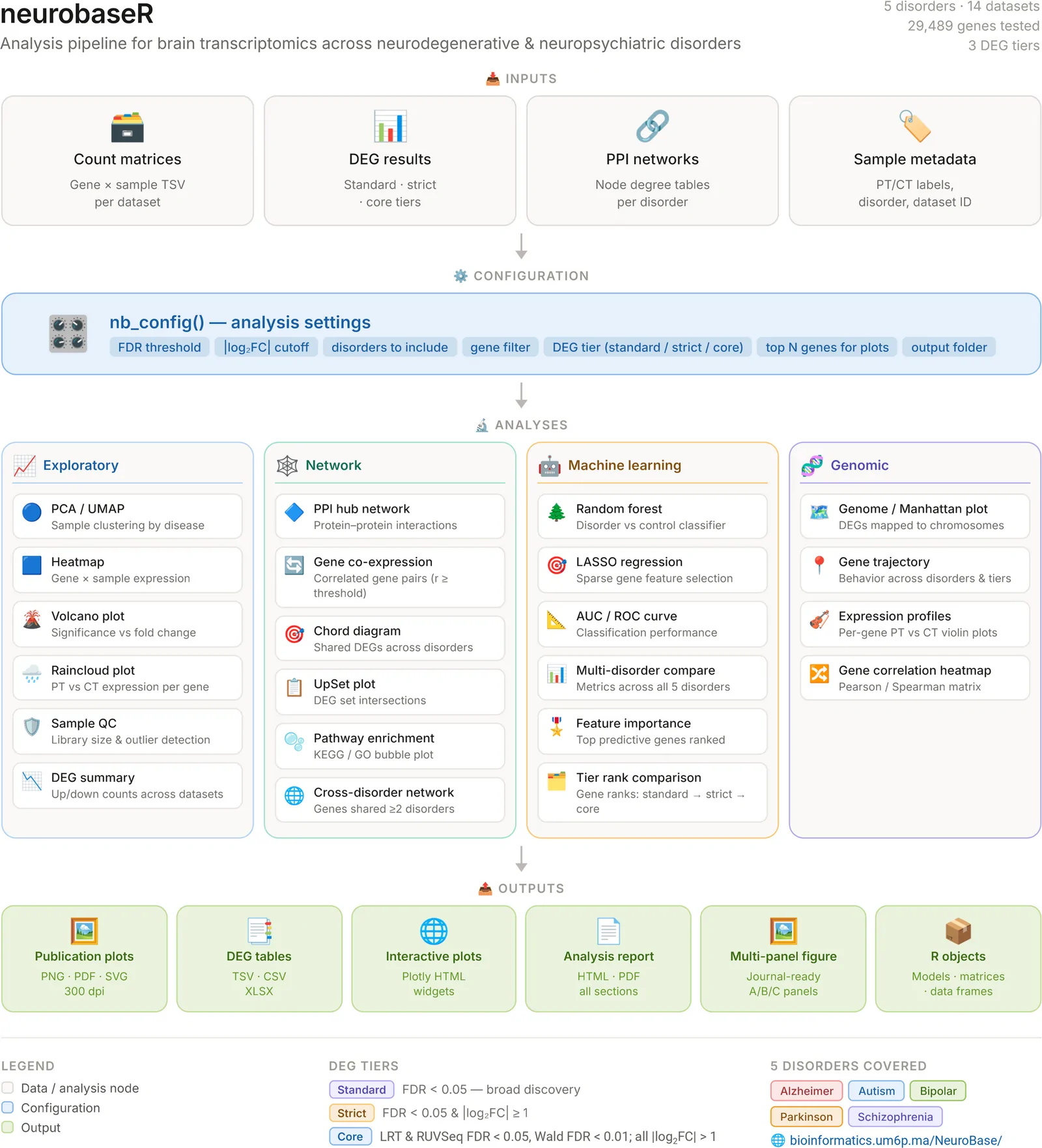
neurobaseR analysis pipeline. The pipeline integrates input data (count matrices, DEG results, PPI networks, and metadata) through configurable parameters, executes four analysis modules (exploratory, network, machine learning, and genomic), and generates publication-ready outputs across three DEG stringency tiers (standard, strict, core) for five neurological disorders (Alzheimer’s, Autism, Bipolar, Parkinson’s, and Schizophrenia). The machine-learning module is an exploratory package utility and was not used for the analyses reported in this article

NeuroBase serves as a valuable tool for accessing and visualizing gene expression data, enabling exploratory analysis and comparative investigation of transcriptomic patterns across neurodegenerative and psychiatric disorders. NeuroBase is freely accessible at: https://bioinformatics.um6p.ma/NeuroBase/ (Fig. 7). To extend programmatic access beyond the web interface, we developed neurobaseR, a companion R package available at https://github.com/AlsammanAlsamman/neurobaseR (Fig. 8). The package provides direct access to all original count data and differential expression results for all 14 datasets, including both the standard (exploratory) and Core Intersection (method-concordant) gene sets described in this study. neurobaseR additionally includes a suite of visualization functions reproducing the key figures available on the web platform, enabling users to generate publication-ready plots within their own R workflows. Together, the web platform and neurobaseR provide complementary access modes — interactive exploration for broad users and scriptable, reproducible analysis for computational researchers.

## Discussion

In our investigation, we identified significantly differentially expressed genes (DEGs) across five neurological disorders (AD, ASD, BD, PD, and SCZ) curated within NeuroBase, and we interpret representative top-ranked signals using published mechanistic evidence. In AD, SPP1 was among the most significant DEGs in our analysis, and human cortical studies associate both SPP1 and ITGAX with cognition and common neuropathologies, supporting a disease-relevant microglial axis (Lopes et al. 2024). Mechanistically, perivascular SPP1 has been shown to drive microglia to engulf synapses in Alzheimer's models, and Spp1 absence prevents this synaptic loss, a finding consistent with our transcriptomic analysis (De Schepper et al. 2023). In PD, NRGN was a top DEG in dataset PRJNA563007 and has been discussed as a synaptic biomarker, mainly in Alzheimer’s disease (Hawksworth et al. 2022), thus positioning NRGN as a candidate for further study in PD within our comparative framework.

Several top-ranked signals have plausible links to known cellular functions. In ASD, BEST1 was among the most significant DEGs and is mechanistically associated with astrocyte-mediated tonic inhibition: channel-mediated tonic GABA release from glia can occur via Bestrophin-1 (Lee et al. 2010), and an astrocyte-specific BEST1 conditional knockout reduces tonic GABA inhibition in vivo (Joo et al. 2024), a circuit-relevant mechanism consistent with excitation/inhibition imbalance frameworks in ASD (Cellot and Cherubini 2014). In BD, FILIP1 was among the most significant DEGs; FILIP regulates cortical neuronal migration out of the ventricular zone (Nagano et al. 2002), and bi-allelic FILIP1 variants cause a recessive disorder with neurological involvement and dysmorphic features (Roos et al. 2023), a link that may be relevant to BD but remains to be tested. Finally, in SCZ, DLX6 was among the most significant DEGs; DLX5/6 programs are essential for the development of parvalbumin-expressing cortical interneurons (Wang et al. 2010), consistent with interneuron dysfunction frameworks implicated in psychiatric disorders (Marín, 2012). Together, these gene-level examples (SPP1, NRGN, BEST1, FILIP1, DLX6) illustrate how NeuroBase connects top-ranked transcriptomic signals to established biological mechanisms spanning microglial synapse remodeling, synaptic integrity, astrocytic inhibitory tone, neurodevelopmental migration, and interneuron specification.

This study provides an in-depth exploration of the molecular and transcriptomic landscape of five major neurodegenerative and neuropsychiatric disorders—AD, ASD, BD, PD, and SCZ, primarily through the development and application of the NeuroBase database. Our findings emphasize both shared and distinct transcriptomic signatures, as represented within the curated transcriptomic profiles available in NeuroBase, offering an exploratory framework for examining molecular features that may underlie these complex conditions.

The GO enrichment outputs available in NeuroBase (not shown in this article) include processes such as inflammatory responses, neurotransmitter receptor transport and neuron-to-neuron synapse, which have been associated with neurodegenerative disorders. Consistent with these database-derived patterns, microglia and astrocytes have been shown to play pivotal roles in regulating inflammatory responses in the central nervous system (Kwon and Koh 2020). The SPP1 gene, which mediates microglial engulfment of synapses in AD models (De Schepper et al. 2023), serves as an illustrative example of how genes highlighted in NeuroBase map onto established inflammatory mechanisms in AD pathology. Similarly, neurotransmitter receptor transport, essential for synaptic function and strength, has been linked to the regulation of synaptic activity in a mouse model Wu et al. (2021). Further, neuron-to-neuron synapse pathways have been reported to be enriched in a mouse model of PD by spatial transcriptomic profiling (Jia et al. 2023), although no PD pathway reached FDR < 0.05 in the present enrichment analysis. These pathway-level insights demonstrate how NeuroBase facilitates the contextualization of transcriptomic patterns within known biological frameworks, rather than serving as direct evidence of causal mechanisms.

Our protein–protein interaction (PPI) analysis within the NeuroBase platform further elucidates patterns of molecular connectivity across disorders. In ASD, TP53 emerged as the most highly connected gene based on STRING network topology, which combines curated, predicted and text-mining evidence and therefore tends to favor well-studied genes. Although TP53 is best known for its role in tumor suppression, it also plays a crucial role in neurodevelopmental disorders (NDDs). Its functions include transcriptional regulation, DNA binding, and other cellular processes essential for neuronal development (Yavuz et al. 2023). While TP53 is rarely mutated in ASD, its recurrent connectivity within the NeuroBase PPI network suggests potential regulatory relevance at the transcriptomic interaction level rather than direct genetic causation. Additionally, STAT3, the second most connected gene in the ASD PPI network, is a crucial regulator of gene expression linked to neuronal development and cell survival (Khera et al. 2022). Together, these observations illustrate how NeuroBase PPI visualizations highlight genes with prominent interaction profiles, supporting exploratory investigation of molecular networks in ASD rather than definitive target identification.

In BD, our study identified AKT1 as a highly interactive gene within the disorder-specific PPI network represented in NeuroBase. AKT1 encodes a serine/threonine kinase implicated in signaling pathways central to neuronal function. Notably, its substrate glycogen synthase kinase-3 (GSK3) is a primary target of lithium, a widely used treatment for BD. These findings are supported by Millischer et al. (2020), who emphasized AKT1 as a vulnerability gene for BD. The prominence of AKT1 in the NeuroBase interaction network provides an example of how known disease-associated genes are reflected in transcriptome-derived PPI structures, reinforcing the utility of the database for contextualizing molecular interactions rather than inferring therapeutic efficacy.

Our PCA analysis as implemented within the NeuroBase exploratory framework revealed clustering patterns that should be interpreted with caution. Because these patient samples were not batch-corrected and each disorder is represented by only two or three datasets, the proximity of AD and ASD and the relative positioning of the other disorders are likely to be driven largely by dataset and tissue effects rather than shared disease biology. The variance observed within AD samples further highlights heterogeneity in transcriptomic profiles across datasets, emphasizing differences in disease representation and sample composition. Overall, these PCA patterns demonstrate how NeuroBase enables exploratory visualization of sample relationships across disorders, supporting comparative analysis rather than definitive mechanistic inference.

Finally, recognizing the need for centralized resources to advance mental health research, we developed NeuroBase as an exploratory, web-based transcriptomic database consolidating data from all five disorders. NeuroBase provides an integrated platform for interactive exploration of gene expression patterns, enriched pathways, and PPI networks, facilitating comparative transcriptomic analysis across disorders rather than direct biological inference. The inclusion of visual tools, such as heatmaps and pathway-based analyses, supports user-driven examination of shared and disorder-specific transcriptomic features. Across disorders, differential expression results are intended as exploratory outputs within NeuroBase, supporting comparative analysis and hypothesis generation rather than confirmatory inference. Shared differentially expressed genes are presented to indicate cross-disorder transcriptomic overlap, while functional pathway interpretation is supported through enrichment analyses available within the NeuroBase platform, enabling users to explore common biological processes such as synaptic and inflammatory pathways. Pathway enrichment results are presented as exploratory summaries derived from DEGs rather than direct measures of pathway activity; therefore, the absence of highlighted pathways in some disorders reflects dataset-specific characteristics rather than definitive lack of pathway involvement.

We acknowledge that bulk RNA-seq lacks cell-type resolution; however, it enables standardized cross-disorder transcriptomic comparison within a single, accessible web-based resource. Differences in sample size across disorders reflect public data availability and may influence statistical power within individual datasets; therefore, the counts of DEG are presented as exploratory, dataset-specific representations rather than comprehensive depictions of disease-associated transcriptional changes. Variability in DEG counts across disorders likely reflects differences in biological heterogeneity, tissue source, sequencing depth, and study-specific technical factors, rather than uniform differences in disease biology. A key methodological consideration in resource-oriented transcriptomic analyses is the balance between sensitivity and specificity in defining significant genes. The primary DEG counts and Venn analyses reported in the Results were derived at FDR ≤ 0.05 in DESeq2 for one dataset per disorder (see Results). This differs from the broader standard tier used for the NeuroBase outputs (significance in at least one of DESeq2, edgeR or limma-voom), which is a deliberate choice to maximize the breadth of discoverable signals available to NeuroBase users. However, we recognize that an FDR threshold without an effect-size filter can inflate DEG counts in heterogeneous datasets, as observed in ASD (6,704 genes) and BD (3,559 genes). To address this, a multi-method sensitivity analysis was performed, yielding Core Intersection gene sets supported by the LRT and RUVSeq-corrected analyses (FDR < 0.05, |log2FC| > 1) and by the Wald test at FDR < 0.01 (see Methods). The substantial reduction in gene counts under stricter criteria — for example, 6,704 DESeq2 DEGs in ASD, compared with 983 method-concordant Core Intersection genes in the harmonized run of the same ASD dataset (PRJNA143369) — reflects expected method-sensitivity in bulk RNA-seq rather than contradictory findings, and underscores the importance of NeuroBase's user-driven filtering design. Researchers seeking method-concordant candidates for experimental follow-up are directed to the Core Intersection gene lists in Supplementary Table S13.

The unified strict-threshold reanalysis further illustrates how stringency and reproducibility interact in cross-disorder transcriptomic comparison (Fig. 2). Two observations are particularly informative. First, the principal component analysis showed that, without batch correction, sample variance is dominated by dataset and tissue origin rather than by disease status; this is an expected property of pooled multi-cohort bulk RNA-seq and underscores why direct cross-study integration must be interpreted cautiously. Second, despite high DEG yield in several individual datasets, only 24 genes were reproducible across two or more independent datasets of the same disorder in a consistent direction, and no gene was reproducible within a single disorder across three or more datasets. This limited reproducibility does not indicate an absence of disease-relevant transcriptomic changes; rather, it reflects the combined effects of small per-study sample sizes, differences in brain region and postmortem handling, and the conservative nature of a per-dataset single-threshold criterion applied without cross-study harmonization. The frequent directional disagreement in cross-disorder gene overlaps (Fig. 2D) reinforces that apparent gene sharing between disorders does not necessarily imply shared regulatory direction. Together, these findings provide the central rationale for NeuroBase's multi-tier reporting strategy: the standard tier maximizes sensitivity for exploratory discovery, the per-dataset strict tier characterizes effect size and directionality within individual cohorts, and the Core Intersection tier (Supplementary Table S13) provides method-concordant candidates for users prioritizing specificity; these candidates still require independent validation. Presenting all three tiers together, rather than collapsing them into a single number, allows users to calibrate analyses to the level of stringency appropriate for their downstream application, from hypothesis generation to candidate gene validation.

In summary, this study introduces NeuroBase as a comprehensive, exploratory transcriptomic resource for neurodegenerative and neuropsychiatric disorders. By integrating standardized gene expression data, pathway enrichment outputs, and protein–protein interaction visualizations within a unified platform, NeuroBase supports comparative analysis and hypothesis generation, providing a foundation for future data-driven research rather than definitive mechanistic or therapeutic conclusions. The companion R package neurobaseR extends this utility by enabling programmatic access to all underlying data, both standard and Core Intersection DEG sets, and integrated visualization functions, making NeuroBase accessible to a broad spectrum of researchers regardless of their preferred analytical environment.

## Supplementary Information

Below is the link to the electronic supplementary material.

Supplementary file1 Differentially expressed genes in Alzheimer's disease (AD); DESeq2 Wald test, FDR < 0.05. (XLSX 6.89 KB)

Supplementary file2 Differentially expressed genes in autism spectrum disorder (ASD); DESeq2 Wald test, FDR < 0.015. (XLSX 259 KB)

Supplementary file3 Differentially expressed genes in bipolar disorder (BD); DESeq2 Wald test, FDR < 0.015. (XLSX 153 KB)

Supplementary file4 Differentially expressed genes in Parkinson's disease (PD); DESeq2 Wald test, FDR < 0.015. (XLSX 24.4 KB)

Supplementary file5 Differentially expressed genes in schizophrenia (SCZ); DESeq2 Wald test, FDR < 0.015. (XLSX 105 KB)

Supplementary file6 Shared and unique DEGs between neurodegenerative (NDs) and neuropsychiatric (NPs) disorders (Venn analysis, FDR < 0.05). (XLSX 8.23 KB)

Supplementary file7 Shared and unique DEGs across all five disorders (AD, ASD, BD, PD, SCZ; Venn analysis, FDR < 0.05). (XLSX 119 KB)

Supplementary file8 Node degree values for the AD protein–protein interaction network (STRING). (XLSX 5.50 KB)

Supplementary file9 Node degree values for the ASD protein–protein interaction network (STRING). (XLSX 31.8 KB)

Supplementary file10 Node degree values for the BD protein–protein interaction network (STRING). (XLSX 20.3 KB)

Supplementary file11 Node degree values for the PD protein–protein interaction network (STRING). (XLSX 8.75 KB)

Supplementary file12 Node degree values for the SCZ protein–protein interaction network (STRING). (XLSX 18.9 KB)

Supplementary file13 Core Intersection differentially expressed genes, per dataset: genes with FDR < 0.05 and |log2FC| > 1 in both the LRT and the RUVSeq-corrected analyses and with FDR < 0.01 and |log2FC| > 1 in the Wald test, a threshold more stringent than the per-dataset strict tier. log2Fold Change is the apeglm-shrunken Wald estimate and padj is the LRT adjusted p-value. These results come from the harmonized pipeline run used for Fig. 2 and Supplementary Table S14, not from the analysis behind Supplementary Tables S1–S7. (XLSX 207 KB)

Supplementary file14 Per-dataset strict-threshold (FDR ≤ 0.05, |log₂FC| ≥ 1) differentially expressed genes for all 14 datasets (S14a) and cross-dataset consensus genes significant in ≥ 2 independent datasets of the same disorder (S14b). (XLSX 774 KB)

Supplementary file15 Volcano plot of differential expression in Alzheimer's disease (AD). x-axis: log₂ fold change (patient vs control); y-axis: −log₁₀ adjusted p-value; significant genes (FDR < 0.05) are highlighted. (PDF 522 kb)

Supplementary file16 Volcano plot of differential expression in autism spectrum disorder (ASD). Axes and thresholds as in Supplementary Fig. S1. (PDF 199 KB)

Supplementary file17 Volcano plot of differential expression in bipolar disorder (BD). Axes and thresholds as in Supplementary Fig. S1. (PDF 112 KB)

Supplementary file18 Volcano plot of differential expression in Parkinson's disease (PD). Axes and thresholds as in Supplementary Fig. S1. (PDF 22.3 KB)

Supplementary file19 Volcano plot of differential expression in schizophrenia (SCZ). Axes and thresholds as in Supplementary Fig. S1. (PDF 392 KB)

## Data Availability

All datasets analyzed in this study are publicly available from the GEO/SRA repositories under the accession numbers listed in Table 1. Processed data, differential expression results across all stringency tiers, pathway enrichment outputs, and protein–protein interaction network data are available through the NeuroBase web platform (https://bioinformatics.um6p.ma/NeuroBase/) and the neurobaseR package (https://github.com/AlsammanAlsamman/neurobaseR).

## Code Availability

The complete analysis pipeline and the source code for the neurobaseR package are available at https://github.com/AlsammanAlsamman/neurobaseR under the MIT license.
