# Supplementary material for: NeuroBase: A Web-Based Transcriptomic Resource and R Package for Cross-Disorder Analysis of Neurodegenerative and Neuropsychiatric Diseases
Source: Mol Neurobiol. 2026 Nov 12;63(1):1028. doi: 10.1007/s12035-026-06169-0 (PMC13759453; doi:10.1007/s12035-026-06169-0)
Supplement: Supplementary file 2 [file 12035_2026_6169_MOESM16_ESM.pdf]

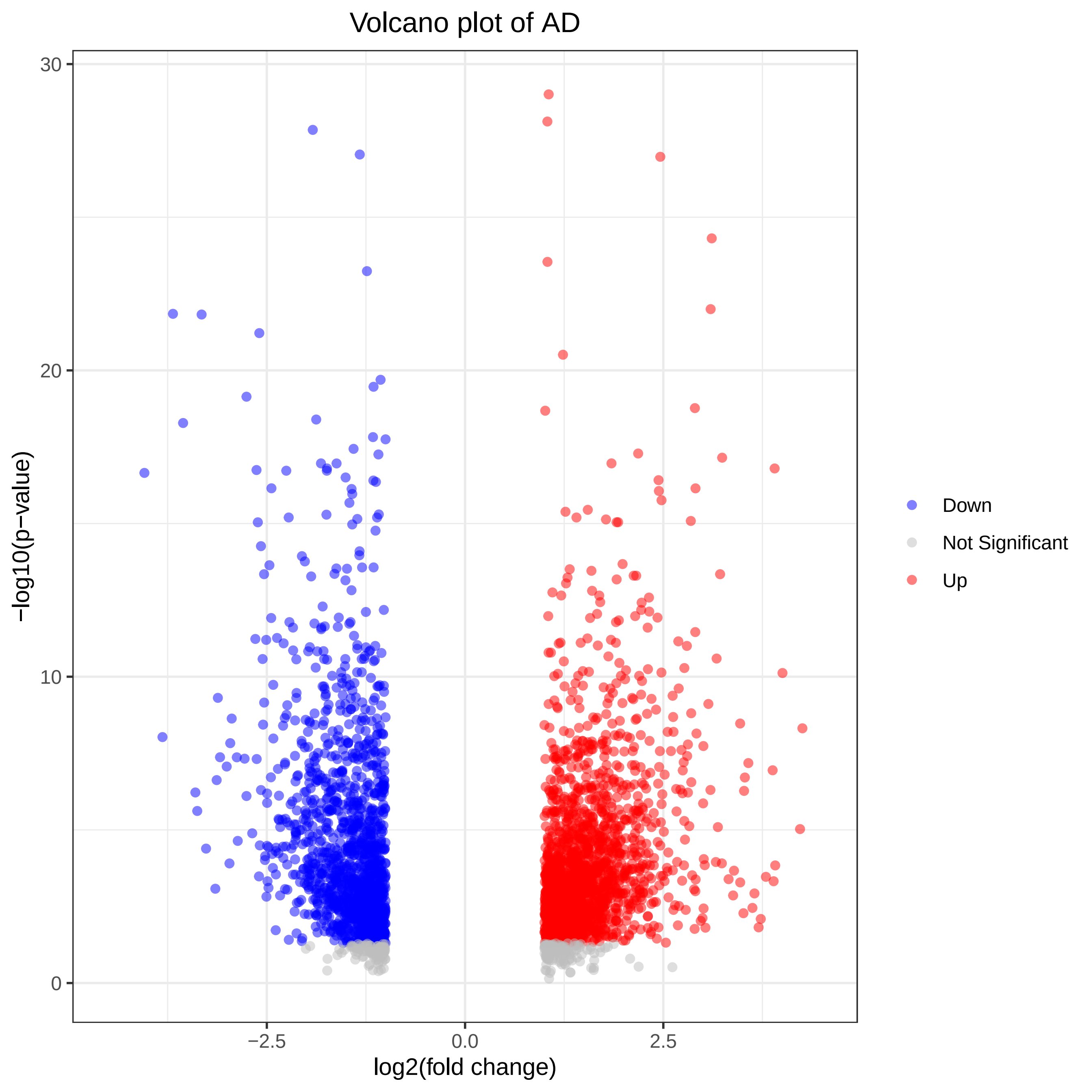

Volcano plot of AD
-log10(p-value)
30
20
10
0
-2.5
0.0
2.5
log2(fold change)
Down
Not Significant
Up
