# Supplementary material for: NeuroBase: A Web-Based Transcriptomic Resource and R Package for Cross-Disorder Analysis of Neurodegenerative and Neuropsychiatric Diseases
Source: Mol Neurobiol. 2026 Nov 12;63(1):1028. doi: 10.1007/s12035-026-06169-0 (PMC13759453; doi:10.1007/s12035-026-06169-0)
Supplement: Supplementary file 3 [file 12035_2026_6169_MOESM17_ESM.pdf]

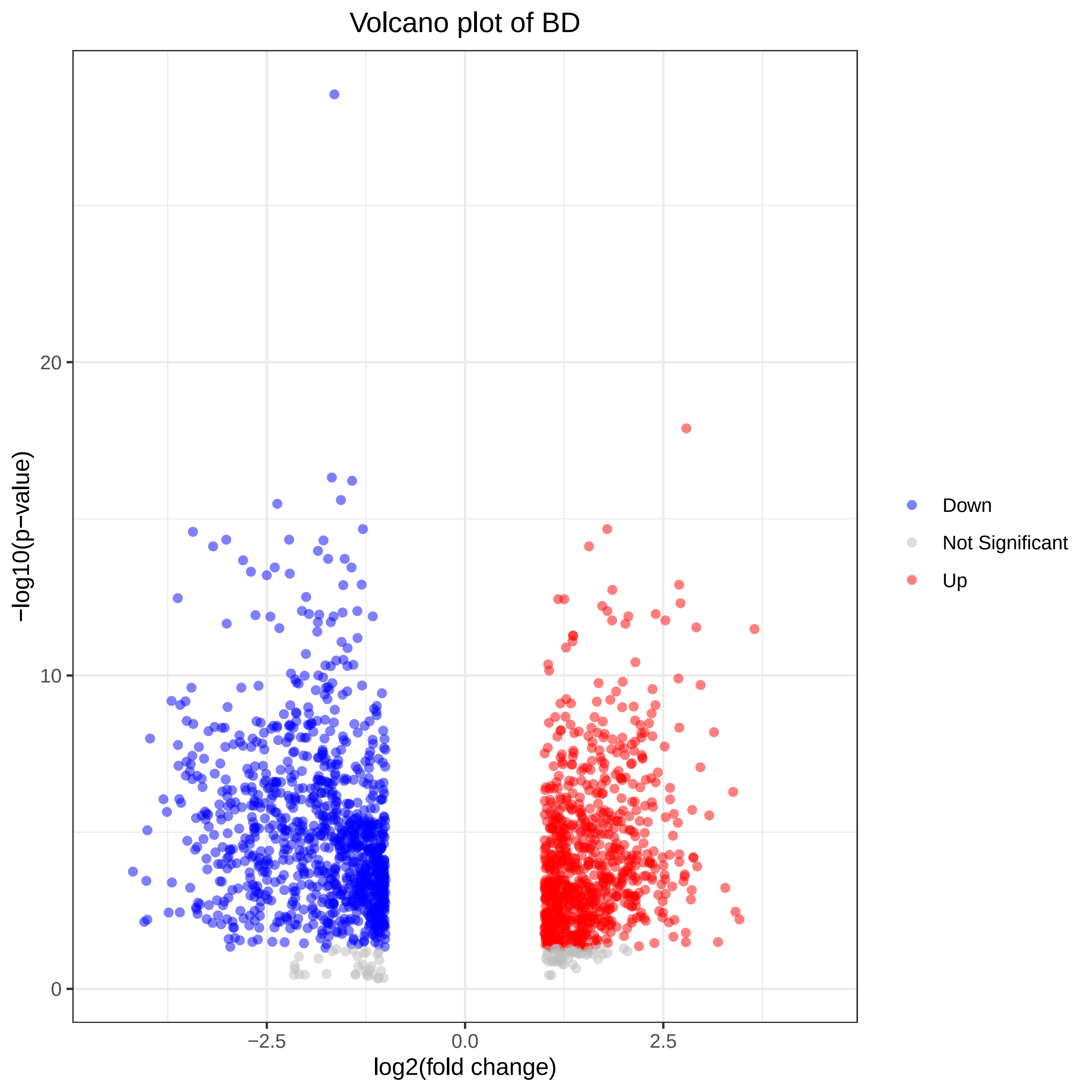
Volcano plot of BD
−log10(p−value)
20
10
0
−2.5
0.0
2.5
log2(fold change)
Down
Not Significant
Up
